# Degenerate Beta autoregressive model for proportion time-series with zeros or ones: An application to antimicrobial resistance rate using R shiny app

**DOI:** 10.3389/fpubh.2022.969777

**Published:** 2023-01-10

**Authors:** Jevitha Lobo, Asha Kamath, Vandana Kalwaje Eshwara

**Affiliations:** ^1^Department of Data Sciences, Prasanna School of Public Health, Manipal Academy of Higher Education (MAHE), Manipal, Karnataka, India; ^2^Department of Microbiology, Kasturba Medical College of Manipal, Manipal Academy of Higher Education (MAHE), Manipal, Karnataka, India

**Keywords:** Beta distribution, time-series model, mixture distribution, rates, proportions, inflated distribution, AMR, resistance

## Abstract

**Background:**

Antimicrobial resistance has emerged as one of the foremost public health troubles of the 21st century. This has ended in a public health disaster of the global situation, which threatens the exercise of present-day remedy. There is an urgent requirement for a cost-effective strategy to reduce antimicrobial resistance. Infectious disease control researchers most often analyze and predict antimicrobial resistance rate data that includes zeros or ones. Commonly used time-series analysis such as autoregressive moving average model is inappropriate for such data and may arrive at biased results.

**Objective:**

This study aims to propose a time-series model for continuous rates or proportions when the interval of series includes zeros or ones and compares the model with existing models.

**Data:**

The *Escherichia coli*, isolated from blood cultures showing variable susceptibility results to different antimicrobial agents, has been obtained from a clinical microbiology laboratory of a tertiary care hospital, Udupi district, Karnataka, during the years between 2011 and 2019.

**Methodology:**

We proposed a Degenerate Beta Autoregressive model which is a mixture of continuous and discrete distributions with probability mass at zero or one. The proposed model includes autoregressive terms along with explanatory variables. The estimation is done using maximum likelihood with a non-linear optimization algorithm. An R shiny app has been provided for the same.

**Results:**

The proposed Degenerate Beta Autoregressive model performed well compared to the existing autoregressive moving average models. The forecasted antimicrobial resistance rate has been obtained for the next 6 months.

**Conclusion:**

The findings of this article could be beneficial to the infectious disease researchers to use an appropriate time-series model to forecast the resistance rate for the future and to have better or advance public health policies to control the rise in resistance rate.

## 1. Introduction

Antimicrobial resistance (AMR) is a serious problem in many developing countries. The World Health Organization (WHO) has categorized AMR as a serious health problem affecting the patients of various countries. In 2015, the WHO surveyed in its six regions called country situation analysis to examine the current practices and to determine the gaps which increase antimicrobial resistance. Eleven and eight countries from Asian and African continents, respectively, from low- to middle-income participated in the study. The analysis showed that AMR is a major issue in both the continents, and in the South-East Asia, nosocomial infections are of particular concern. The main cause of resistance is the inappropriate use of antimicrobial medicines and poor healthcare facilities (1).

In the past, to study antibiotic usage and resistance time-series, models such as Box-Jenkins ARIMA (autoregressive-integrated moving average) (2) and transfer function models have been used. Some studies used time-series to forecast trend and seasonality of the data with the exponential smoothing technique.

However, there is a limitation of these models in terms of accuracy of prediction or violation of assumptions in the applicability. Hence, this study proposes to develop and test stochastic models to predict antibiotic resistance rate, which helps us in understanding the pattern of resistance to plan strategies for the rational use of antibiotics.

Oftentimes, the data like antimicrobial resistance include data in the interval [0, 1) or (0, 1], when the bacteria is highly susceptible or resistant to the antibiotic. To model data with rates/proportions regression, models are proposed by Ferrari and Cribari-Neto (3), Mitnik and Baek (4), Pumi et al. (5), and Artur and Bazán (6) and the time-series models are proposed by Rocha and Cribari-Neto (7) and Bayer et al. (8). But these models are not appropriate for the proportion data with zeros or ones. To model such kind of data, we look for a mixture of distributions. Ospina and Ferrari (9) and Cribari-Neto and Santos (10) introduced inflated Beta distributions and inflated Kumaraswamy distributions which is a mixture of discrete and continuous distributions. Ospina and Ferrari (11) and Bayer et al. (12) introduced the inflated Beta regression model and inflated Kumaraswamy regression model. Currently, there is scope to use a time-series model for proportion data in the interval [0, 1) or (0, 1].

This paper proposes to model the time-series data in the interval [0, 1) or (0, 1] using a mixture of Degenerate and Beta distributions through a frequentist approach. This study is an extension of *β*ARMA model proposed by Rocha and Cribari-Neto (7), where instead of Beta distribution, inflated Beta distribution is incorporated. The proposed model is compared with the ARIMA model.

The developed model is illustrated with an application based on antimicrobial resistance (AMR) data. Rates of *Escherichia coli* (*E. coli*) isolated from blood cultures showing variable susceptibility results to different antimicrobial agents are considered for the modeling in this study. *E. coli* is a gram-negative bacteria, most regularly isolated in patients with blood stream infection (BSI), and in severe instances, it may cause loss of life. The rates of BSI have accelerated steadily in recent years (13). However, knowledge of future resistance rate using forecasting may help in recommending new interventions or policy recommendations in hospital settings.

## 2. Materials and methods

### 2.1. Data description

The variable susceptibility of *Escherichia coli* (*E. coli*), isolated from blood cultures showing variable susceptibility results to different antimicrobial agents, has been obtained from a clinical microbiology laboratory of a tertiary care hospital, Udupi district, Karnataka, during the years between 2011 and 2019. Institutional ethical clearance was obtained from Kasturba Medical College and Kasturba Hospital Institutional Ethics Committee (IEC no. 832/2019). The laboratory generally receives more than 10,000 blood culture tests annually from patients who have been suspected of bacteremia/sepsis. The blood cultures yielded positive results in 20–30% of cases and *E. coli* is the most common among several other bacteria causing bacteremia in our patient population. We retrieved the data of antimicrobial susceptibility test (AST) results of this bacteria from the electronic records of the laboratory. Antimicrobial susceptibility tests were performed using the Kirby Bauer disc diffusion method until 2014 and by the Vitek-2 automated method after 2015, both of which are accepted and standardized test methods according to the Clinical Laboratory Standards Institute (Ref:CLSI supplement M100. Wayne, PA: Clinical and Laboratory Standards Institute; 2012). In the former method, the bacteria was considered resistant to the antimicrobial agent amoxicillin-clavulanic acid, based on the ESBL phenotypic test, although the individual agent appeared susceptible. However, in the latter method, results were reported unchanged as detected in the automated system. Laboratory had tested *E. coli* against different antimicrobial agents and the results were interpreted as either susceptible or resistant. The data include information on the monthly number of *E. coli* isolates obtained and the total number of isolates resistant to the different antibiotics which includes a total of 108 time points. The resistance proportion is calculated as the number of *E. coli* isolates that were resistant to a particular antimicrobial agent/total number of *E. coli* tested during that time interval. This study considered the data on the antibiotic amoxicillin-clavulanic acid (AMC) and Cefoperazone-sulbactam (CSL) since it met the requirements of the model (i.e., time dependency and in the interval [0,1) or (0,1]).

### 2.2. Degenerate Beta autoregressive (De*β*AR) model

#### 2.2.1. Beta distribution

The parameterized probability density function of random variable X of Beta distribution is

(1) $$\begin{array}{l} f\left(x;\mu ,\zeta \right)=\frac{\Gamma \left(\zeta \right)}{\Gamma \left(\mu \zeta \right)\Gamma \left(\left(1-\mu \right)\zeta \right)}x^{\mu \zeta -1}{\left(1-x\right)}^{\left(1-\mu \right)\zeta -1},0<x<1 \end{array}$$

Here, 0 < μ < 1 and ζ > 0. The mean and variance of the random variable X is, *E*(*X* = *x*) = μ and $V\left(X=x\right)=\frac{V\left(\mu \right)}{1+\zeta }$; where *V*(μ) = μ(1 − μ). Hence, here μ and ζ act as distribution mean and precision parameters. Here, when value of μ is fixed, as the value of ζ increases, the variance of x decreases.

#### 2.2.2. Degenerate distribution

A Degenerate distribution is a one-point distribution where a random variable X has a single possible value.

The probability mass function of random variable X can be written as follows:

(2) $$\begin{array}{l} P\left(X=x\right)=\{\begin{array}{ll} 1 & \text{if }x=c;\\ 0 & \text{elsewhere} \end{array} \end{array}$$

That is, a random variable, X, is degenerate if, for some constant, c, P(X = c) = 1.

This distribution has a single parameter, c, and it ranges from - ∞ to ∞.

#### 2.2.3. Inflated Beta distribution

Ospina and Ferrari (9) introduced inflated Beta distributions which is a mixture of Degenerate and Beta distributions. The probability density function is

(3) $$\begin{array}{l} bi_{c}\left(x;\omega ,\mu ,\zeta \right)=\{\begin{array}{ll} \omega & \text{if }x=c;\\ \left(1-\omega \right)f\left(x;\mu ,\zeta \right) & \text{if }x\in \text{(0,1)} \end{array} \end{array}$$

where, 0 < ω < 1 is the mixture parameter, *f*(*x*; μ, ζ) is the p.d.f of Beta distribution and c=0 or 1 known value which follows Degenerate distribution. The cumulative distribution function (c.d.f) of mixture distribution is given by

$$BI_{c}\left(x;\omega ,\mu ,\zeta \right)=\omega I_{\left[c,1\right]}\left(x\right)+\left(1-\omega \right)F\left(x;\mu ,\zeta \right)$$

Where, $I_{A}\left(x\right)$ is an indicator function that equals 1 if *x* ∈ *A* and 0 if *x* ∉ *A*. Here, *F*(.; μ, ζ) is the c.d.f. of the beta distribution.

The *r*^*th*^ moment and variance of inflated beta distribution is


$$E\left(x^{r}\right)=\omega c+\left(1-\omega \right)\mu_{r}$$

$$Var\left(x\right)=\left(1-\omega \right)\frac{V\left(\mu \right)}{\zeta +1}+\omega \left(1-\omega \right){\left(c-\mu \right)}^{2}$$

where, μ_*r*_ = (μζ)_*r*_/ζ_*r*_.

#### 2.2.4. Beta autoregressive moving average model

Rocha and Cribari-Neto (7) introduced *β*ARMA model to fit continuous time-series data in the interval (0, 1), which follows Beta distribution.

The proposed *β*ARMA(p, q) model is

(4) $$\begin{array}{l} g\left(\mu_{t}\right)=\beta_{0}+s_{t}^{\prime }\beta +\Sigma_{i=1}^{p}\phi_{i}\left\{g\left(x_{t-i}\right)-s_{t-i}^{\prime }\beta \right\}+\Sigma_{j=1}^{q}\alpha_{j}r_{t-j} \end{array}$$

where, g(.) is the link function, β_0_ is the intercept term, *s*_*t*_'s are the regressor variables, and $\beta ={\left(\beta_{1},\beta_{2},\ldots ,\beta_{k}\right)}^{\prime }$ are set of parameters of regressors. The ϕ's and the α's are the autoregressive (AR) and moving average (MA) parameters, p and q are the AR and MA orders, and *r*_*t*_ is an error term, respectively.

In case of a real-life scenario when time-series data includes zeros or ones, it is challenging to use this model by assuming Beta distribution. To model such kind of continuous time-series data, we replaced Beta distribution with inflated Beta distribution proposed by Ospina and Ferrari (9). The density function of inflated Beta distribution with time can be written as follows:

Let *X*_*t*_ t=1,2…n be the response variable of proportion data which includes zeros or ones. We assume that the proportion series is conditionally distributed as InBE (μ_*t*_, ζ, ω) (where, InBE stands for "Inflated Beta") with probability density function defined as:

(5) $$\begin{array}{l} f_{X_{t}}\left(x_{t}|F_{t-1}\right)=\omega I_{\left(x_{t}=c\right)}+\left(1-\omega \right)\frac{\Gamma \left(\zeta \right)}{\Gamma \left(\mu_{t}\zeta \right)\Gamma \left(\left(1-\mu_{t}\right)\zeta \right)}\\ x_{t}^{\mu_{t}\zeta -1}{\left(1-x_{t}\right)}^{\left(1-\mu_{t}\right)\zeta -1} \end{array}$$

or equivalently

$$\begin{array}{l} f_{X_{t}}\left(x_{t}|F_{t-1}\right)\\ =\begin{array}{l} \{\omega \;ifx=c;\\ \left(1-\omega \right)\frac{\Gamma \left(\zeta \right)}{\Gamma \left(\mu_{t}\zeta \right)\Gamma \left(\left(1-\mu_{t}\right)\zeta \right)}x_{t}^{\mu_{t}\zeta -1}{\left(1-x_{t}\right)}^{\left(1-\mu_{t}\right)\zeta -1}\;ifx\in \left(0,1\right) \end{array} \end{array}$$

which is a mixture of Beta and Degenerate distributions. Here, $F_{t-1}$ is the previous information set of response series. When the mixture parameter ω = 0, inflated Beta distribution reduces to Beta distribution. Based on Equations (3) and (4), the mean and variance of distribution can be written as follows:

For [0,1),

$$E\left(X_{t}|F_{t-1}\right)=\left(1-\omega \right)\mu_{t}$$

$$V\left(X_{t}|F_{t-1}\right)=\left(1-\omega \right)\frac{\mu_{t}\left(1-\mu_{t}\right)}{\zeta +1}+\left\{\omega \left(1-\omega \right)\mu_{t}^{2}\right\}$$

For (0,1],

$$E\left(X_{t}|F_{t-1}\right)=\omega +\left(1-\omega \right)\mu_{t}$$

$$V\left(X_{t}|F_{t-1}\right)=\left(1-\omega \right)\frac{\mu_{t}\left(1-\mu_{t}\right)}{\zeta +1}+\left\{\omega \left(1-\omega \right){\left(1-\mu_{t}\right)}^{2}\right\}$$

#### 2.2.5. Proposed model

The proposed Degenerate Beta Autoregressive (De*β*AR) model for the parameters of mixture distribution is

(6) $$\begin{array}{l} \eta_{t}=g\left(\mu_{t}\right)=s_{t}^{\prime }\beta +\Sigma_{i=1}^{p}\phi_{i}\left\{g\left(x_{t-i}\right)-s_{t-i}^{\prime }\beta \right\} \end{array}$$

where, η_*t*_ is the linear or non-linear predictor of the model, where g(.) is the link function (we used logit), *s*_*t*_'s are the regressor variables, and $\beta ={\left(\beta_{1},\beta_{2},\ldots ,\beta_{k}\right)}^{\prime }$ are the unkonwn parameters of regressor variables. $\phi_{i}={\left(\phi_{1},\phi_{2},\ldots ,\phi_{p}\right)}^{\prime }$ are the autoregressive parameters with order p. Let $\theta ={\left(\beta^{\prime },\zeta ,\omega ,\phi_{i}^{\prime }\right)}^{\prime }$ be vector of unknown parameters with length k+p+2.

#### 2.2.6. Parameter estimation

The parameters of the model are estimated by maximizing log-likelihood function.

Here, let *X*_*t*_, t=1,2,…,n be a random variable and $F_{t-1}$ be the set of past information. Then, the likelihood function of the parameters θ conditioning on the past p observations can be written as follows:


$$\begin{array}{l} L\left(\theta ;x_{t}\right)=\overset{n}{\underset{t=p+1}{\prod }}f_{X_{t}}\left(x_{t}|F_{t-1}\right) \end{array}$$

$$\begin{array}{l} L\left(\theta ;x_{t}\right)=\overset{n}{\underset{t=p+1}{\prod }}bi_{c}\left(z_{t};\omega ,\mu_{t},\zeta \right)=L_{1}\left(\omega \right)L_{2}\left(\mu_{t},\zeta \right) \end{array}$$

where,


$$\begin{array}{l} L_{1}\left(\omega \right)=\overset{n}{\underset{t=p+1}{\prod }}\omega^{I_{x_{t}=c}}{\left(1-\omega \right)}^{I_{x_{t}\epsilon \left(0,1\right)}} \end{array}$$

$$\begin{array}{r} L_{2}(\mu_{t},\zeta )=\prod_{t=p+1}^{n}\{\frac{\Gamma (\zeta )}{\Gamma (\mu_{t}\zeta )\Gamma ((1-\mu_{t})\zeta )}x_{t}^{\mu_{t}\zeta -1}(1-x_{t})\\ {\;}^{\;(1-\mu_{t})\zeta -1}{\}}^{{ℐ}_{x_{t}\epsilon (0,1)}} \end{array}$$

The likelihood function for the parameters of Degenerate Beta AR model is given by,

$$\begin{array}{r} L(\theta ;x_{t})=\prod_{t=p+1}^{n}\{\omega {ℐ}_{x_{t}=c}+{ℐ}_{x_{t}\epsilon (0,1)}(1-\omega )\\ \frac{\Gamma (\zeta )}{\Gamma (\mu_{t}\zeta )\Gamma ((1-\mu_{t})\zeta )}x_{t}^{\mu_{t}\zeta -1}(1-x_{t}{)}^{(1-\mu_{t})\zeta -1}\} \end{array}$$

Then, the log-likelihood of model is

$$\begin{array}{l} log\left(L\left(\theta ;x\right)\right)=l\left(\theta \right)\\ =\overset{n}{\underset{t=p+1}{\sum }}I_{x_{t}=c}log\left(\omega \right)+\overset{n}{\underset{t=p+1}{\sum }}I_{x_{t}\epsilon \left(0,1\right)}log\{\left(1-\omega \right)\\ \frac{\Gamma \left(\zeta \right)}{\Gamma \left(\mu_{t}\zeta \right)\Gamma \left(\left(1-\mu_{t}\right)\zeta \right)}x_{t}^{\mu_{t}\zeta -1}{\left(1-x_{t}\right)}^{\left(1-\mu_{t}\right)\zeta -1}\} \end{array}$$

Here, take $I_{x_{t}=c}=x_{ct}$, then *l*(***θ***) equals to

$$\begin{array}{l} \overset{n}{\underset{t=p+1}{\sum }}x_{ct}log\left(\omega \right)+\overset{n}{\underset{t=p+1}{\sum }}\left(1-x_{ct}\right)log\{\left(1-\omega \right)\\ \frac{\Gamma \left(\zeta \right)}{\Gamma \left(\mu_{t}\zeta \right)\Gamma \left(\left(1-\mu_{t}\right)\zeta \right)}x_{t}^{\mu_{t}\zeta -1}{\left(1-x_{t}\right)}^{\left(1-\mu_{t}\right)\zeta -1}\} \end{array}$$

Then, the score function is given by

$$U\left(\theta \right)=\frac{\partial }{\partial \theta }l\left(\theta \right)=0$$

i.e., for l=1,2…k

$$\frac{\partial l\left(\theta \right)}{\partial \beta_{l}}=\overset{n}{\underset{t=p+1}{\sum }}\frac{\partial l\left(\theta \right)}{\partial \mu_{t}}\frac{\partial \mu_{t}}{\partial \eta_{t}}\frac{\partial \eta_{t}}{\partial \beta_{l}}$$

Note that, $\frac{\partial \mu_{t}}{\partial \eta_{t}}=\mu_{t}\left(1-\mu_{t}\right)$ and $\frac{\partial \eta_{t}}{\partial \beta_{l}}=s_{tl}-\overset{p}{\underset{i=1}{\sum }}\phi_{i}s_{\left(t-i\right)l}$.

Then,

$$\begin{array}{l} \frac{\partial l\left(\theta \right)}{\partial \beta_{l}}=\overset{N}{\underset{t=p+1}{\sum }}\left(1-x_{ct}\right)\zeta [log\left(\frac{x_{t}}{1-x_{t}}\right)\\ -\left\{\psi \left(\mu_{t}\zeta \right)-\psi \left(\left(1-\mu_{t}\right)\zeta \right)\right\}]\mu_{t}\left(1-\mu_{t}\right)\left(s_{tl}-\overset{p}{\underset{i=1}{\sum }}\phi_{i}s_{\left(t-i\right)l}\right) \end{array}$$

Here, let $x_{t}^{*}=log\left(\frac{x_{t}}{1-x_{t}}\right)$ if *x*_*t*_ϵ(0, 1) else $x_{t}^{*}=0$ (11, 14) and $\psi \left(\mu_{t}\zeta \right)-\psi \left(\left(1-\mu_{t}\right)\zeta \right)=\mu_{t}^{*}$, where ψ(.) is a digamma function. Then,

$$\frac{\partial l\left(\theta \right)}{\partial \beta_{l}}=\overset{n}{\underset{t=p+1}{\sum }}\left(1-x_{ct}\right)\zeta \left(x_{t}^{*}-\mu_{t}^{*}\right)\mu_{t}\left(1-\mu_{t}\right)\left(s_{tl}-\overset{p1}{\underset{i=1}{\sum }}\phi_{i}s_{\left(t-i\right)l}\right)$$

Similarly,

$$\begin{array}{l} \frac{\partial l\left(\theta \right)}{\partial \zeta }=\overset{n}{\underset{t=p+1}{\sum }}\zeta \left(1-x_{ct}\right)\{\mu_{t}\left(x_{t}^{*}-\mu_{t}^{*}\right)+log\left(1-x_{t}\right)\\ -\psi \left(\left(1-\mu_{t}\right)\zeta \right)+\psi \left(\zeta \right)\} \end{array}$$

$$\frac{\partial l\left(\theta \right)}{\partial \omega }=\overset{n}{\underset{t=p+1}{\sum }}\frac{\left(x_{ct}-\omega \right)}{\omega \left(1-\omega \right)}$$

For i=1,2…p

$$\frac{\partial l\left(\theta \right)}{\partial \phi_{i}}=\overset{n}{\underset{t=p+1}{\sum }}\left(1-x_{ct}\right)\zeta_{t}\left(x_{t}^{*}-\mu_{t}^{*}\right)\mu_{t}\left(1-\mu_{t}\right)\left(g\left(x_{t-i}\right)-s_{t-i}^{\prime }\beta \right)$$

The maximum likelihood estimator of ***θ*** is obtained by equating *U*(***θ***) = 0. Since there exists no closed form solution for these equations, a non-linear optimization algorithm like Newton's method or a Quasi-Newton algorithm such as limited-memory Broyden–Fletcher–Goldfarb–Shanno algorithm (L-BFGS-B) has been used (15, 16).

Practically, we can use the gamlss function in R software in the package GAMLSS (generalized additive models for location scale and shape) (17) to get the initial values for estimating the parameters.

In this study, we have used the Quasi-Newton method (18) under which we performed the L-BFGS-B algorithm to obtain the optimum solution for the parameters.

Large sample inference: If the model specified by Equation (5) follows the regularity condition of maximum likelihood estimation (MLE) then, MLE of ***θ*** and *J*(***θ***) (Fisher information matrix) are consistent. Assuming that $I\left(\theta \right)=\lim_{n\to \infty }\left\{n^{-1}J\left(\theta \right)\right\}$ exists and is nonsingular, we have $\sqrt{n}\left(\hat{\theta }-\theta \right)$ converges in distribution to *N*(0, *I*(***θ***)^−1^).

Note: The proposed DeβAR model is applicable when $x_{t}^{*}$ is converted to 0 as mentioned above. To overcome with this limitation Bayer et al. (19) proposed Inflated beta autoregressive moving average models, which are more suitable when interval data includes 0 or 1.

### 2.3. Simulation study

In this simulation study, we featured finite-sample performance of the MLE. Due to time consumption, the simulated time-series data generated only for De*β*AR with lag 1 and lag 2.

$$\eta_{t}=logit\left(\mu_{t}\right)=\beta_{0}+\phi_{1}g\left(x_{t-1}\right)$$

$$\eta_{t}=logit\left(\mu_{t}\right)=\beta_{0}+\phi_{1}g\left(x_{t-1}\right)+\phi_{2}g\left(x_{t-2}\right)$$

Here, ζ and ω are constant for all observations. We took β_0_ = 1.2, ϕ_1_ = −0.8, ϕ_2_ = −0.2, ζ = 50, and ω = 0.5 as true parameters.

The Monte Carlo simulation with 15,000 replications was carried out each with sample size *n* = 100, 250, and 500. Of the 15,000 replicants, 5,000 were the burn out. Parameter estimates were obtained as the convergent outcome of the remaining 10,000. The parameters are estimated by maximizing the log-likelihood function using the L-BFGS-B algorithm. The bias and root mean square error of the estimates are reported.

Table 1 represents the bias and root mean square error of the parameters. Here, we can observe that, as the sample size increases, the algorithm converged for all the samples. The mean of β_0_, ϕ_1_, ϕ_2_, and ω are close to the true values or the initial values. Also, the root mean square errors of all the estimators are decreased as the size of the sample increases, as anticipated.

**Table 1:** Simulation results.

	**Sample size (n)**	**100**		**250**		**500**	
**Model**	**Estimators**	**Bias**	$\sqrt{MSE}$	**Bias**	$\sqrt{MSE}$	**Bias**	$\sqrt{MSE}$
	β_0_	0.001	0.057	0.0006	0.0364	0.0001	0.026
D*β*AR(1)	ϕ_1_	–0.002	0.072	–0.001	0.045	–0.0007	0.031
	ζ	–3.222	12.603	–1.308	6.883	–0.637	4.679
	ω	0.003	0.206	–0.0004	0.032	–0.00007	0.022
	β_0_	0.005	0.110	0.0004	0.048	0.0008	0.034
	ϕ_1_	–0.002	0.125	–0.0006	0.053	–3.77E–05	0.037
D*β*AR(2)	ϕ_2_	-0.008	0.134	–0.002	0.055	-0.001	0.039
	ζ	–10.306	26.772	–1.758	7.159	–0.881	4.712
	ω	0.0003	0.073	–0.0003	0.032	–0.0001	0.022

### 2.4. Forecast evaluation criteria

Selecting a proper model among several competing candidates is a hassle in a lot of time-series analysis. In many cases, to look for out-of-sample forecast accuracy, the MAPE (“Mean absolute percentage error") is used for model selection or comparison. However, when data are close to zero, other forecast measurement criteria can be used, such as MAE (“mean absolute error"), MSE (“mean square error"), RMSE (“root mean square error"), and ex post forecast error (20). In the case of time-series analysis, the ACF plot is one of the model selection criteria, and the AIC (“Akaike information criterion") and the BIC (“Bayesian information criterion") will be used for an in-sample accuracy check.

### 2.5. R shiny application

R shiny is a website application available freely to build under R-studio. It is an interactive website application that requires no web development skills^1^. R shiny web app can be accessed at DeBAR.app^2^.

## 3. Results

### 3.1. Exploratory data analysis

**Figure 1:**
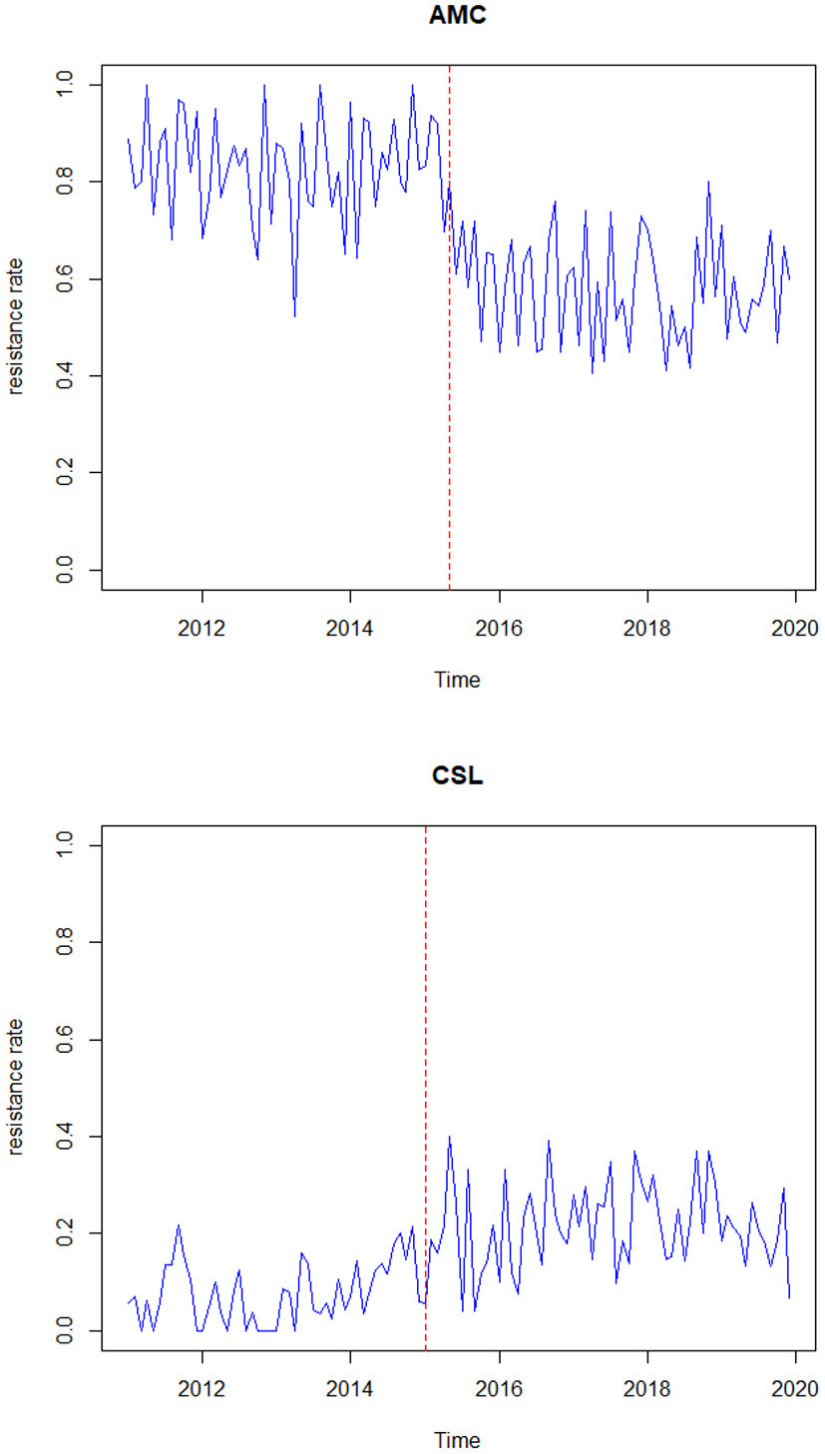
Time plot of proportion of *E. coli* in blood resistant to antibiotics AMC and CSL.

**Figure 2:**
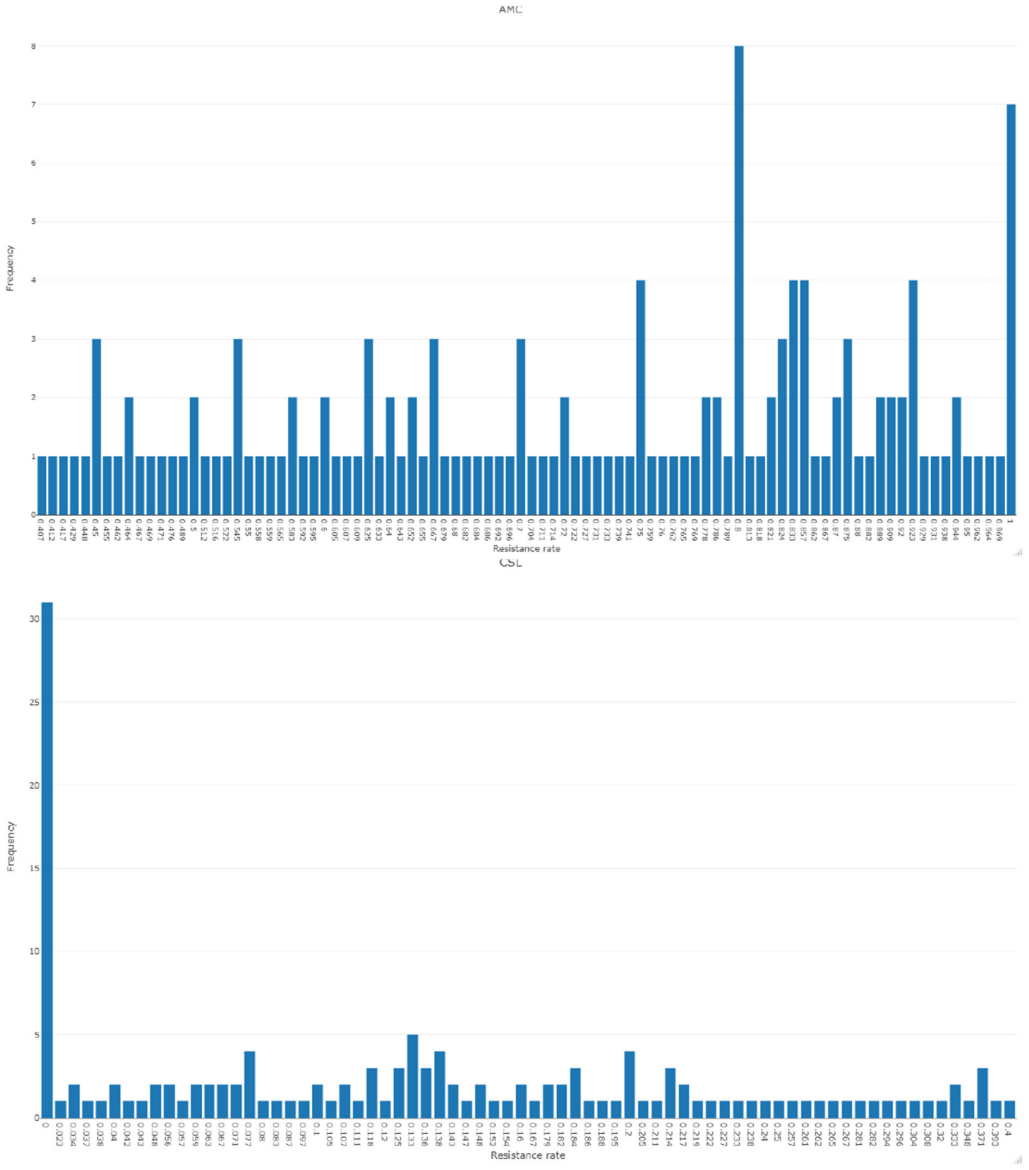
Histogram of proportion of *E. coli* in blood resistant to antibiotics AMC and CSL.

To understand the characteristics of the response series, the descriptive statistics for the data are represented in Table 2. Figure 1 represents the time-series plot of the bacteria *E. coli* isolated from blood cultures resistant to the antimicrobial agents AMC and CSL, where the sudden change between the years 2015 and 2016 is due to the improvisation in the testing method in April 2015, which involved a shift from manual testing method (i.e., Kirby Bauer disc diffusion method) to Vitek-2 automated method from the year 2015. This change point has been considered as a covariate in the model and represented as an indicator variable *I*_*t*_. Figure 2 displays the histogram of the same. The proposed model is applicable to model stationary time-series data. The ACF plot will be used to identify the stationarity in the series. If non-stationarity exists, then significant deterministic elements can be added as a regressor variable in the model. As the current study data include change points in the series, stationarity cannot be identified through the ACF plot.

**Table 2:** Descriptive statistics for the data on rate of *E. coli* resistant to AMC and CSL.

**Antibiotic**	**Sample size**	**Range**	**Mean**	**Std.Deviation**	**Skewnes**	**Min**	**Max**	**Median**
AMC	108	0.593	0.703	0.165	–0.0024	0.407	1	0.702
CSL	108	0.4	0.15	0.10	0.40	0	0.4	0.14

### 3.2. Time-series analysis

The data have been modeled using Degenerate Beta Autoregressive (De*β*AR(p)) model proposed in Section 2 and the analysis has been carried out using the software R. Quasi-Newton algorithm (L-BFGS-B) has been used to maximize the partial log-likelihood function.

First, the following De*β*AR(p) models, where p=1, 2, 3,…,12 fitted to the AMC data,

$$Model1:\eta_{t}=logit\left(\mu_{t}\right)=\beta_{0}+\alpha I_{t}+\phi_{1}\left\{g\left(x_{t-1}\right)\right\}$$

$$Model2:\eta_{t}=logit\left(\mu_{t}\right)=\beta_{0}+\alpha I_{t}+\Sigma_{i=1}^{2}\phi_{i}\left\{g\left(x_{t-i}\right)\right\}$$

$$Model3:\eta_{t}=logit\left(\mu_{t}\right)=\beta_{0}+\alpha I_{t}+\Sigma_{i=1}^{3}\phi_{i}\left\{g\left(x_{t-i}\right)\right\}$$

$$Model12:\eta_{t}=logit\left(\mu_{t}\right)=\beta_{0}+\alpha I_{t}+\Sigma_{i=1}^{12}\phi_{i}\left\{g\left(x_{t-i}\right)\right\}$$

where, *x*_*t*−*i*_ i=1,2,…,12 is the lagged response series and *I*_*t*_ is the change point (Indicator) variable with coefficient α. Among the 12 models, the best model is selected using the AIC and the BIC for which these values are minimum. The selected model is then compared with the existing ARIMA model.

The AIC and the BIC select Model 1 as the best among the 12 models as its values are minimum compared to the others (**Table 4**). From the significance test (Table 3), it can be seen that for the data AMC, lag-1 is significant at 5% level of significance (l.o.s) and for data CSL, lag-1 is significant at 10% level of significance (l.o.s) along with the indicator variable and parameters of the model, respectively. Next, the model is compared with the ARIMA model. Autocorrelation function (ACF) plots for residuals, forecast accuracy criteria (MAE, MSE, and MAPE), and information criteria (AIC and BIC) were used to select the best model for the given data.

**Table 3:** Fitted De*β*AR model for rate of *E. coli* resistant to AMC and CSL.

**Antibiotic**	**Parameter**	**Estimate**	**Std. Error**	**z stat**	***p*** **-value**
	β_0_	1.689	0.156	10.808	0.00*e*^+00^
	ϕ_1_	–0.109	0.080	–1.367	1.74*e*^−02^
AMC	*log*(ρ)	2.798	0.554	5.043	2.15*e*^−06^
	*logit*(Ω)	–3.188	0.099	–32.043	0.00*e*^+00^
	α	–1.338	0.150	–8.889	3.59*e*^−14^
	β_0_	–2.185	0.166	–13.112	0.00e+00
	ϕ_1_	–0.0215	0.068	–0.313	7.547e-02
CSL	*log*(ρ)	3.099	0.020	148.21	0.00e+00
	*logit*(Ω)	–2.102	0.319	–6.580	2.46e-09
	α	0.877	0.128	6.834	7.55e-10

Models considered were,

Model 1: De*β*AR(p), with lag at 1

$$\eta_{t}=logit\left(\mu_{t}\right)=\beta_{0}+\alpha I_{t}+\phi_{1}g\left(x_{t-1}\right)$$

Model 2: ARIMA(0, 0, 1) model using Arcsine transformation

$$y_{t}=\beta_{0}+\alpha I_{t}-\theta_{1}\epsilon_{t-1}$$

In the analysis, as a final step, data have been forecasted by keeping out the last six observations (hold-out data) from the time-series, and Model 1 and Model 2 have fitted to the selected (test data) series and forecasted for the next 6 months. The out-of-sample forecast accuracy are calculated using the hold-out and forecasted data from the models and comparisons between models have been carried out. The hold-out series of AMC is 0.545, 0.592, 0.7, 0.469, 0.667, and 0.6. The forecasted series from Model 1 is 0.597, 0.594, 0.6, 0.595, 0.595, and 0.6. The forecasted series from Model 2 is 0.602, 0.579,0.579, 0.579, 0.579, and 0.579. Similarly, the hold-out series of CSL is 0.205, 0.184, 0.133, 0.184, 0.294, and 0.067. The forecasted series from Model 1 is 0.192, 0.194, 0.193, 0.193, 0.194, and 0.195. The forecasted series from Model 2 is 0.223, 0.215, 0.213, 0.214, 0.214, and 0.213.

**Figure 3:**
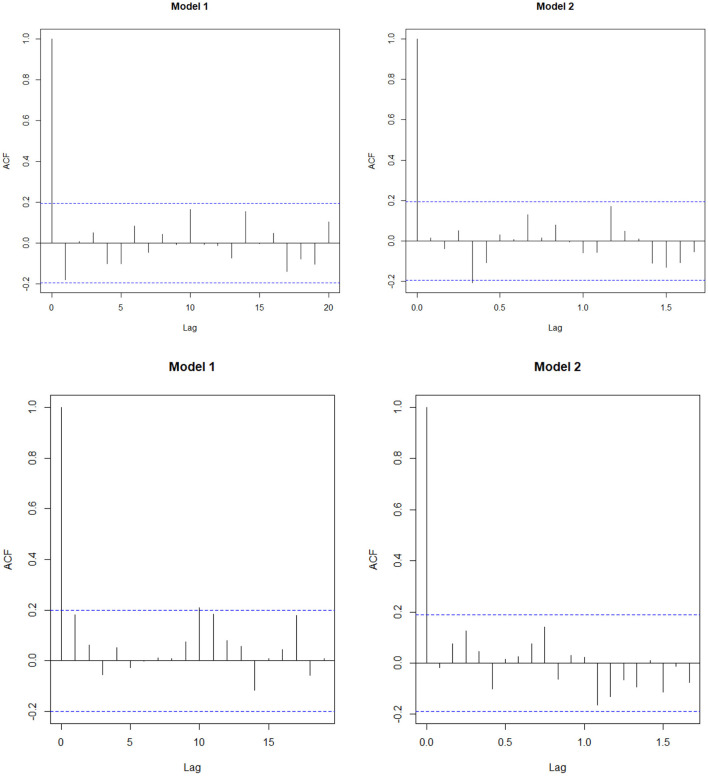
Sample ACF of the residuals obtained from the fitted models for AMC and CSL.

The study found that for both the data, the forecast accuracy for Model 1 is better compared to the Model 2 (**Table 5**) and residual plot of Model 1 follows white noise assumptions (i.e., the residual series should have mean 0 and no autocorrelation within the series), whereas for AMC data (Figure 3), Model 2 is serially correlated at lag 4. Thus, we select Model 1 as the best fit model for both the data.

Thus, the estimated De*β*AR(1) model for AMC is

$$\hat{\mu_{t}}=\frac{exp\left(\beta_{0}+\alpha I_{t}+\phi_{1}g\left(x_{t-1}\right)\right)}{1+exp\left(\beta_{0}+\alpha I_{t}+\phi_{1}g\left(x_{t-1}\right)\right)}$$

$$E\left(x\right)=\hat{\omega }+\left(1-\hat{\omega }\right)\hat{\mu_{t}}$$

where, $\left(\hat{\beta_{0}},\hat{\alpha },\hat{\phi_{1}},\hat{\omega }\right)$ = (1.68, –1.34, –0.11, 0.03). The forecasted series for next 6 months is 0.604, 0.587, 0.613, 0.587, 0.604, and 0.582.

Similarly, the estimated De*β*AR(1) model for CSL is

$$\hat{\mu_{t}}=\frac{exp\left(\beta_{0}+\alpha I_{t}+\phi_{1}g\left(x_{t-1}\right)\right)}{1+exp\left(\beta_{0}+\alpha I_{t}+\phi_{1}g\left(x_{t-1}\right)\right)}$$

$$E\left(x\right)=\left(1-\hat{\omega }\right)\hat{\mu_{t}}$$

where, $\left(\hat{\beta_{0}},\hat{\alpha },\hat{\phi_{1}},\hat{\omega }\right)$ = (–2.185,0.877, –0.021, 0.108). The forecasted series for next 6 months is 0.19, 0.20, 0.19, 0.194, 0.192, and 0.189.

The R code of the analysis has been provided in the supporting file.

## 4. Discussion

AMR is difficult to control and expensive to deal with, and the outcome is that it can lead to death or severe disability. Many researchers have used different statistical techniques to look at the relationship between antimicrobial use and resistance. To list a few of them, a study by Athanasiou and Kopsini (21) in 2018 systematically reviewed the statistical methods used to analyze the AMR rate time-series data. Many of the studies used the ARIMA model (22) and the transfer function model and few studies used the multiple linear regression model. Infectious disease control researchers most often analyze and predict antimicrobial resistance rate data, which includes zeros or ones [resistance rate with zeros can be seen in the figure of the article by Lopez-Lozano et al. (23)]. The commonly used ARIMA model is inappropriate for non-Gaussian (not normally distributed) time-series data and may arrive at biased results. Rocha and Cribari-Neto (7) and Bayer et al. (8) introduced the Beta autoregressive moving average model and the Kumaraswamy autoregressive moving average model to fit rate/proportion time-series data in the interval (0, 1). To analyze proportion data with zeros or ones, Ospina and Ferrari (11) proposed an inflated beta regression model, and as an alternative, Bayer et al. (12) proposed an inflated Kumaraswamy regression model. But none of the studies addressed the autocorrelation in the response series in the interval [0, 1) or (0, 1]. So, to fill this gap, in this article, we proposed the Degenerate Beta Autoregressive (De*β*AR) model.

For this intention, we collected time-series data on *E*.*coli* isolates resistant to the different antimicrobial agents, and for the current study, only amoxicillin–clavulanic acid (AMC) and Cefoperazone–sulbactam (CSL) were considered. The primary focus of this study is to forecast the AMR rate for the time-series data in the interval (0, 1] or [0, 1). The proposed De*β*AR(p) models (where p=1,2,…,12) fitted to the data and best order for “p” was selected for which the values of the AIC and the BIC were minimum. The best model among these was then compared with the existing ARIMA model and the best among both was decided based on the forecasting evaluation criteria (MSE, MAE, and MAPE).

From Table 4, we can see that for the data AMC, the values of the AIC and the BIC are minimum for Model 1 (i.e., AIC = −126.19 and BIC = −112.78) compared to other models. The study found that the De*β*AR (1) model performed well compared to the remaining 11 models. Next, the selected De*β*AR(1) model is compared with the existing ARIMA (0, 0, 1) model selected from the autogeneration. From Table 5, we can see the values of MAE, MSE, and MAPE are minimum for the proposed Model 1 compared to the existing Model 2 along with the AIC and BIC. Hence, the study selected the De*β*AR(1) model as the best among all. Similar procedure followed for the data of CSL.

**Table 4:** AIC and BIC for D*β*AR(p) models for AMC and CSL data.

**Antibiotic**	**AMC**		**CSL**	
**Model**	**AIC**	**BIC**	**AIC**	**BIC**
Model 1	–126.19	–112.78	–153.69	–140.28
Model 2	–121.92	–105.83	–149.96	–133.86
Model 3	–117.64	–98.86	–152.58	–133.81
Model 4	–122.33	–100.87	–147.01	–125.55
Model 5	–119.55	–95.42	–151.37	–127.23
Model 6	–115.17	–88.35	–146.66	–119.84
Model 7	–110.99	–81.48	–142.14	–112.63
Model 8	–108.40	–76.22	-140.54	–108.35
Model 9	–105.93	–71.07	-138.07	–103.21
Model 10	–110.76	–73.22	–134.39	–96.84
Model 11	–106.62	–66.39	–133.56	–93.32
Model 12	–103.46	–60.55	–136.87	–93.96

**Table 5:** Model selection criterion.

**Antibiotic**	**Model**	**Log-likelihood**	**MAE**	**MSE**	**MAPE**	**AIC**	**BIC**
AMC	Model 1	68.09	0.089	0.011	13.93	–126.19	–112.78
	Model 2	57.81	0.109	0.018	18.40	–107.62	–97.12
CSL	Model 1	75.64	0.053	0.005	—	–153.69	–140.28
	Model 2	63.39	0.064	0.006	—	–118.79	–108.29

By using the proposed De*β*AR(1) model, the AMR rate was forecasted for next 6 months. The study results indicate the forecasted resistance rate of *E. coli* to the antimicrobial AMC ranges between 58 and 62% for the next 6 months, implying constant variations in the resistance rate.

The De*β*AR(p) model introduced in this article would be beneficial to healthcare providers to implement early public health measures to control and prevent the rise in resistance rate.

## 5. Conclusion and future direction

Antimicrobial resistance is an emerging issue of public health. However, taking appropriate precautions or interventions in advance may help in reducing the resistance rate. Forecasting using an appropriate time-series model may help in predicting the expected resistance rate for the future which is further useful for policy-making.

This study proposed the Degenerate Beta Autoregressive model (De*β*AR) to model antimicrobial resistance rate data, which is an extension of the *β*ARMA model proposed by Rocha and Cribabri-Neto (2009). The proposed model can be used to fit continuous time-series data in the interval [0, 1) and (0, 1], for example, rates or proportions. The model is applicable when the series is stationary in nature. The parameters of the model are estimated by maximizing the likelihood function and closed-form solutions for the score function are obtained by using the non-linear optimization algorithm (L-BFGS-B). The application of the model is presented using AMR data.

The outcome from a time-series model helps the healthcare policymakers to implement an appropriate intervention in advance to reduce the risk of rise in resistance rate.

In future, the study results can be improvised by considering antimicrobial consumption as a regressor variable and the proposed model can be improvised by incorporating moving average terms and seasonal components.

## Data availability statement

The original contributions presented in the study are included in the article/Supplementary material, further inquiries can be directed to the corresponding author.

## Ethics statement

The studies involving human participants were reviewed and approved by Institutional Ethical Clearance was obtained from Kasturba Medical College and Kasturba Hospital Institutional Ethics Committee (IEC No. 832/2019). Written informed consent for participation was not required for this study in accordance with the national legislation and the institutional requirements.

## Author contributions

JL, AK, and VK framed the objective of the study. VK collected the data. JL conducted the analysis, drafted initial manuscript, and developed R shiny app. AK and VK revised the manuscript. All authors have read and approved the final manuscript.

## Footnotes

^1^ https://shiny.rstudio.com/

^2^ https://jevithalobo.shinyapps.io/column/
