# Corrigendum: Degenerate Beta autoregressive model for proportion time-series with zeros or ones: an application to antimicrobial resistance rate using R shiny app

**DOI:** 10.3389/fpubh.2024.1415866

**Published:** 2024-05-16

**Authors:** Jevitha Lobo, Asha Kamath, Vandana Kalwaje Eshwara

**Affiliations:** ^1^Department of Data Sciences, Prasanna School of Public Health, Manipal Academy of Higher Education (MAHE), Manipal, Karnataka, India; ^2^Department of Microbiology, Kasturba Medical College of Manipal, Manipal Academy of Higher Education (MAHE), Manipal, Karnataka, India

**Keywords:** Beta distribution, time-series model, mixture distribution, rates, proportions, inflated distribution, AMR, resistance

In the published article reference 14 was not cited in the article and an additional citation for reference 11 was missed. The citations have now been inserted in **Material and Methods**, ***Degenerate Beta Autoregressive*** ***(DeβAR)*** ***model***, ***Parameter estimation*** and should read:

“Here, let ${\text{x}}_{t}^{*}=log\left(\frac{x_{t}}{1-x_{t}}\right)$ if *x*_*t*_ϵ(0, 1) else ${\text{x}}_{t}^{*}=0$ (11, 14) and $\psi \left(\mu_{t}\zeta \right)-\psi \left(\left(1-\mu_{t}\right)\zeta \right)=\mu_{t}^{*}$, where ψ(.) is a digamma function.”

In the published article, there was an error. Inbetween steps of likelihood derivation was missed.

A correction has been made to **Material and Methods**, ***Degenerate Beta Autoregressive*** ***(DeβAR)*** ***model***, ***Parameter estimation***. “This sentence previously stated:”


$$L\left(\theta ;x\right)=\overset{n}{\underset{t=p+1}{\prod }}f_{X_{t}}\left(x_{t}|F_{t-1}\right)$$

The likelihood function for the parameters of Degenerate Beta AR model is given by,


$$L\left(\theta ;x\right)=\overset{n}{\underset{t=p+1}{\prod }}\left\{\omega I_{x_{t}=c}+I_{x_{t}\epsilon \left(0,1\right)}\left(1-\omega \right)\frac{\Gamma \left(\zeta \right)}{\Gamma \left(\mu_{t}\zeta \right)\Gamma \left(\left(1-\mu_{t}\right)\zeta \right)}x_{t}^{\mu_{t}\zeta -1}{\left(1-x_{t}\right)}^{\left(1-\mu_{t}\right)\zeta -1}\right\}$$

“The corrected sentence appears below:”


$$\begin{array}{l} L\left(\theta ;x_{t}\right)=\overset{n}{\underset{t=p+1}{\prod }}f_{X_{t}}\left(x_{t}|F_{t-1}\right) \end{array}$$

$$\begin{array}{l} L\left(\theta ;x_{t}\right)=\overset{n}{\underset{t=p+1}{\prod }}bi_{c}\left(z_{t};\omega ,\mu_{t},\zeta \right)=L_{1}\left(\omega \right)L_{2}\left(\mu_{t},\zeta \right) \end{array}$$

where,


$$\begin{array}{l} L_{1}\left(\omega \right)=\overset{n}{\underset{t=p+1}{\prod }}\omega^{I_{x_{t}=c}}{\left(1-\omega \right)}^{I_{x_{t}\epsilon \left(0,1\right)}} \end{array}$$

$$\begin{array}{r} L_{2}(\mu_{t},\zeta )=\prod_{t=p+1}^{n}\{\frac{\Gamma (\zeta )}{\Gamma (\mu_{t}\zeta )\Gamma ((1-\mu_{t})\zeta )}x_{t}^{\mu_{t}\zeta -1}(1-x_{t})\\ {\;}^{\;(1-\mu_{t})\zeta -1}{\}}^{{ℐ}_{x_{t}\epsilon (0,1)}} \end{array}$$

The likelihood function for the parameters of Degenerate Beta AR model is given by,


$$\begin{array}{r} L(\theta ;x_{t})=\prod_{t=p+1}^{n}\{\omega {ℐ}_{x_{t}=c}+{ℐ}_{x_{t}\epsilon (0,1)}(1-\omega )\\ \frac{\Gamma (\zeta )}{\Gamma (\mu_{t}\zeta )\Gamma ((1-\mu_{t})\zeta )}x_{t}^{\mu_{t}\zeta -1}(1-x_{t}{)}^{(1-\mu_{t})\zeta -1}\} \end{array}$$

In the published article, there was an error. Limitation of the model has been added and reference 19 has been added.

A correction has been made to **Material and Methods**, ***Degenerate Beta Autoregressive*** ***(DeβAR)*** ***model***, ***Parameter estimation***. “This sentence previously stated:”

Large sample inference: If the model specified by Equation (5) follows the regularity condition of maximum likelihood estimation (MLE) then, MLEs of ***θ*** and *J*(***θ***) (Fisher information matrix) are consistent. Assuming that $I\left(\theta \right)=\underset{n\to \infty }{\lim}\left\{n^{-1}J\left(\theta \right)\right\}$ exists and is non-singular, we have $\sqrt{n}\left(\hat{\theta }-\theta \right)$ converges in distribution to *N*(0, *I*(***θ***)^−1^).

“The corrected sentence appears below:”

Large sample inference: If the model specified by **Equation (5)** follows the regularity condition of maximum likelihood estimation (MLE) then, MLE of ***θ*** and *J*(***θ***) (Fisher information matrix) are consistent. Assuming that $I\left(\theta \right)=\underset{n\to \infty }{\lim}\left\{n^{-1}J\left(\theta \right)\right\}$ exists and is nonsingular, we have $\sqrt{n}\left(\hat{\theta }-\theta \right)$ converges in distribution to *N*(0, *I*(***θ***)^−1^).

Note: The proposed DeβAR model is applicable when $x_{t}^{*}$ is converted to 0 as mentioned above. To overcome with this limitation Bayer et al. (19) proposed Inflated beta autoregressive moving average models, which are more suitable when interval data includes 0 or 1.

The authors apologize for this error and state that this does not change the scientific conclusions of the article in any way. The original article has been updated.
