# Supplementary material for: Degenerate Beta autoregressive model for proportion time-series with zeros or ones: An application to antimicrobial resistance rate using R shiny app
Source: Front Public Health. 2023 Jan 10;10:969777. doi: 10.3389/fpubh.2022.969777 (PMC9873260; doi:10.3389/fpubh.2022.969777)
Supplement: Supplementary file 1 [file Data_Sheet_1.PDF]

Supplementary Material

1 THE INFORMATION MATRIX (I.E., NEGATIVE HESSIAN) OF THE EXβAR

$$H(\boldsymbol{\theta}) = -\frac{\partial^2}{\partial\boldsymbol{\theta}\partial\boldsymbol{\theta'}} logPL(\boldsymbol{\theta})$$

i.e,

$$\frac{\partial^2 l(\boldsymbol{\theta})}{\partial\boldsymbol{\beta_l}^2} = \sum_{t=p+1}^{n} \frac{\partial}{\partial\mu_t}(\frac{\partial l(\boldsymbol{\theta})}{\partial\mu_t}\frac{\partial\mu_t}{\partial\eta_t}\frac{\partial\eta_t}{\partial\boldsymbol{\beta_l}})\frac{\partial\mu_t}{\partial\eta_t}\frac{\partial\eta_t}{\partial\boldsymbol{\beta_l}}$$

Then,
$h_{11} = \frac{\partial^2 l(\boldsymbol{\theta})}{\partial\boldsymbol{\beta_l}^2} = \sum_{t=p+1}^{n}(1-x_{ct})\{\zeta\frac{\partial\mu_t^*}{\partial\mu_t}(\frac{\partial\mu_t}{\partial\eta_t}) - \zeta(x_t^* - \mu_t^*)(1-2\mu_t)\}\frac{\partial\mu_t}{\partial\eta_t}(\frac{\partial\eta_t}{\partial\boldsymbol{\beta_l}})^2$
$h_{12} = h_{21} = \frac{\partial^2 l(\boldsymbol{\theta})}{\partial\boldsymbol{\zeta}\partial\boldsymbol{\beta_l}} = \sum_{t=p+1}^{n}(1-x_{ct})\{\zeta\frac{\partial\mu_t^*}{\partial\zeta_t} - (x_t^* - \mu_t^*)\}\frac{\partial\mu_t}{\partial\eta_t}\frac{\partial\eta_t}{\partial\boldsymbol{\beta_l}}$
$h_{13} = h_{31} = \frac{\partial^2 l(\boldsymbol{\theta})}{\partial\boldsymbol{\omega}\partial\boldsymbol{\beta_l}} = 0$
$h_{14} = h_{41} = \frac{\partial^2 l(\boldsymbol{\theta})}{\partial\boldsymbol{\phi_i}\partial\boldsymbol{\beta_l}} = \sum_{t=p+1}^{n}(1-x_{ct})\{\zeta\frac{\partial\mu_t^*}{\partial\mu_t}(\frac{\partial\mu_t}{\partial\eta_t}) - \zeta(x_t^* - \mu_t^*)(1-2\mu_t)\}\frac{\partial\mu_t}{\partial\eta_t}\frac{\partial\eta_t}{\partial\boldsymbol{\beta_l}}\frac{\partial\eta_t}{\partial\boldsymbol{\phi_i}}$
$h_{22} = \frac{\partial^2 l(\boldsymbol{\theta})}{\partial\boldsymbol{\zeta}^2} = \sum_{t=p+1}^{n}(1-x_{ct})[\{\mu_t\frac{\partial\mu_t^*}{\partial\zeta} + \psi'((1-\mu_t)\zeta)(1-\mu_t) - \psi'(\zeta)\}\frac{\partial\zeta}{\partial\tau_t} - \{\mu_t(x_t^* - \mu_t^*) + log(1-$
$x_t) - \psi((1-\mu_t)\zeta) + \psi(\zeta)\}]$
$h_{23} = h_{32} = \frac{\partial^2 l(\boldsymbol{\theta})}{\partial\boldsymbol{\zeta}\partial\boldsymbol{\omega}} = 0$
$h_{24} = h_{42} = \frac{\partial^2 l(\boldsymbol{\theta})}{\partial\boldsymbol{\zeta}\partial\boldsymbol{\phi_i}} = \sum_{t=p+1}^{n}(1-x_{ct})\{\zeta\frac{\partial\mu_t^*}{\partial\zeta} - (x_t^* - \mu_t^*)\}\frac{\partial\mu_t}{\partial\eta_t}\frac{\partial\eta_t}{\partial\boldsymbol{\phi_i}}$
$h_{33} = \frac{\partial^2 l(\boldsymbol{\theta})}{\partial\boldsymbol{\omega}^2} = \frac{\omega(2x_{ct}-\omega)-x_{ct}}{\omega^2(1-\omega)^2}$
$h_{34} = h_{43} = \frac{\partial^2 l(\boldsymbol{\theta})}{\partial\boldsymbol{\omega}\partial\boldsymbol{\phi_i}} = 0$
$h_{44} = \frac{\partial^2 l(\boldsymbol{\theta})}{\partial\boldsymbol{\phi_i}^2} = \sum_{t=p+1}^{n}(1-x_{ct})\{\zeta\frac{\partial\mu_t^*}{\partial\mu_t}(\frac{\partial\mu_t}{\partial\eta_t}) - \zeta(x_t^* - \mu_t^*)(1-2\mu_t)\}\frac{\partial\mu_t}{\partial\eta_t}(\frac{\partial\eta_t}{\partial\boldsymbol{\phi_i}})^2$

2 THE FISHER INFORMATION MATRIX OF THE EXβAR

$$J(\boldsymbol{\theta}) = E(\frac{\partial^2}{\partial\boldsymbol{\theta}\partial\boldsymbol{\theta'}} logPL(\boldsymbol{\theta}))$$

i.e,

$$j_{11} = E(\frac{\partial^2 l(\boldsymbol{\theta})}{\partial\boldsymbol{\beta_l}^2}|\mathcal{F}_{t-1}) = \sum_{t=p+1}^{n} E(\frac{\partial^2 l(\boldsymbol{\theta})}{\partial\mu_t^2}|\mathcal{F}_{t-1})(\frac{\partial\mu_t}{\partial\eta_t})^2(\frac{\partial\eta_t}{\partial\boldsymbol{\beta_l}})^2$$

Then,
$j_{11} = \frac{\partial^2 l(\boldsymbol{\theta})}{\partial\boldsymbol{\beta_l}^2} = \sum_{t=p+1}^{n}(1-x_{ct})\zeta_t\frac{\partial\mu_t^*}{\partial\mu_t}(\frac{\partial\mu_t}{\partial\eta_t})^2(\frac{\partial\eta_t}{\partial\boldsymbol{\beta_l}})^2$
$j_{12} = j_{21} = E(\frac{\partial^2 l(\boldsymbol{\theta})}{\partial\boldsymbol{\zeta}\partial\boldsymbol{\beta_l}}) = \sum_{t=p+1}^{n}(1-x_{ct})\zeta\frac{\partial\mu_t^*}{\partial\zeta}\frac{\partial\mu_t}{\partial\eta_t}\frac{\partial\eta_t}{\partial\boldsymbol{\beta_l}}$
$j_{13} = j_{31} = E(\frac{\partial^2 l(\boldsymbol{\theta})}{\partial\boldsymbol{\gamma_l}\partial\boldsymbol{\beta_l}}) = 0$
$j_{14} = j_{41} = E(\frac{\partial^2 l(\boldsymbol{\theta})}{\partial\boldsymbol{\phi_i}\partial\boldsymbol{\beta_l}}) = \sum_{t=p+1}^{n}(1-x_{ct})\zeta\frac{\partial\mu_t^*}{\partial\mu_t}(\frac{\partial\mu_t}{\partial\eta_t})^2\frac{\partial\eta_t}{\partial\boldsymbol{\beta_l}}\frac{\partial\eta_t}{\partial\boldsymbol{\phi_i}}$

$j_{22} = E(\frac{\partial^2 l(\boldsymbol{\theta})}{\partial \boldsymbol{\zeta}^2}) = \sum_{t=p+1}^{n} (1 - x_{ct})[\{\mu_t \frac{\partial \mu_t^*}{\partial \zeta} + \psi'((1-\mu_t)\zeta)(1-\mu_t) - \psi'(\zeta)\} + log(1-x_t) - \psi((1 - \mu_t)\zeta) + \psi(\zeta)\}]$

$j_{23} = j_{32} = E(\frac{\partial^2 l(\boldsymbol{\theta})}{\partial \boldsymbol{\zeta} \partial \boldsymbol{\omega}}) = 0$

$j_{24} = j_{42} = E(\frac{\partial^2 l(\boldsymbol{\theta})}{\partial \boldsymbol{\zeta} \partial \boldsymbol{\phi_i}}) = \sum_{t=p+1}^{n} (1 - x_{ct}) \zeta \frac{\partial \mu_t^*}{\partial \zeta} \frac{\partial \mu_t}{\partial \eta_t} \frac{\partial \eta_t}{\partial \boldsymbol{\phi_i}}$

$j_{33} = E(\frac{\partial^2 l(\boldsymbol{\theta})}{\partial \boldsymbol{\omega}^2}) = \frac{\omega(2x_{ct} - \omega) - x_{ct}}{\omega^2 (1-\omega)^2}$

$j_{34} = j_{43} = E(\frac{\partial^2 l(\boldsymbol{\theta})}{\partial \boldsymbol{\omega} \partial \boldsymbol{\phi_i}}) = 0$

$j_{44} = E(\frac{\partial^2 l(\boldsymbol{\theta})}{\partial \boldsymbol{\phi_i}^2}) = \sum_{t=p+1}^{n} (1 - x_{ct}) \zeta_t \frac{\partial \mu_t^*}{\partial \mu_t} (\frac{\partial \mu_t}{\partial \eta_t})^2 (\frac{\partial \eta_t}{\partial \boldsymbol{\phi_i}})^2$
